# Substitution of I222L-E119V in neuraminidase from highly pathogenic avian influenza H7N9 virus exhibited synergistic resistance effect to oseltamivir in mice

**DOI:** 10.1038/s41598-021-95771-4

**Published:** 2021-08-11

**Authors:** Jing Tang, Rongbao Gao, Liqi Liu, Shuxia Zhang, Jia Liu, Xiyan Li, Qiongqiong Fang, Zhaomin Feng, Cuiling Xu, Weijuan Huang, Dayan Wang

**Affiliations:** National Institute for Viral Disease Control and Prevention, Chinese Center for Disease Control and Prevention; WHO Collaborating Center for Reference and Research on Influenza, No.155 Changbai Road, Changping District, Beijing, China

**Keywords:** Microbiology, Molecular biology

## Abstract

That the high frequency and good replication capacity of strains with reduced susceptibility to neuraminidase inhibitors (NAIs) in highly pathogenic avian influenza H7N9 (HPAI H7N9) virus made it a significance to further study its drug resistance. HPAI H7N9 viruses bearing NA I222L or E119V substitution and two mutations of I222L-E119V as well as their NAIs-sensitive counterpart were generated by reverse genetics for NA inhibition test and replication capability evaluation in vitro. The attenuated H7N9/PR8 recombinant viruses were developed to study the pathogenicity and drug resistance brought by the above substitutions to mice. The IC_50_ fold change of oseltamivir to HPAI H7N9 with NA222L-119V is 306.34 times than that of its susceptible strain, and 3.5 times than the E119V mutant virus. HPAI H7N9 bearing NA222L-119V had good replication ability with peak value of more than 6log_10_ TCID_50_/ml in MDCK cells. H7N9/PR8 virus bearing NA222L-119V substitutions leaded to diffuse pneumonia, significant weight loss and fatality in mice. NA E119V made H7N9/PR8 virus resistant to oseltamivir, and I222L-E119V had synergistic resistance to oseltamivir in mice. Due to the good fitness of drug resistant strains of HPAI H7N9 virus, it is necessary to strengthen drug resistance surveillance and new drug research.

## Introduction

Human infection with highly pathogenic avian influenza (HPAI) H7N9 virus appeared at the end of 2016 and re-emerged at 2019^1–3^. As the mutant of H7N9, HPAI H7N9 virus is characterized by the insertion of polybasic amino acids at the cleavage of its hemagglutinin (HA) protein^4,5^. Although the mortality of HPAI H7N9 virus in laboratory confirmed human cases is not significantly higher to that of its low pathogenic (to birds) counterpart, the former seems to have a shorter course of disease and more severe clinical symptoms^6^.

Neuraminidase inhibitors (NAIs) are the only way to treat influenza infection in most countries in the world currently^7^. The approved NAIs in China, included oseltamivir, zanamivir and peramivir, and most H7N9 patients received oseltamivir^1,8–10^. Laninamivir, as well as the polymerase inhibitor baloxavir marboxil, which were recently approved by some countries/areas, are possible antiviral options against influenza infection^11–13^. Due to the poor error correction ability of the single stranded RNA virus^14^, influenza virus is prone to adaptive mutation for survival^15^. Early use of appropriate NAIs can prevent mice infected with lethal dose of H7N9 virus from severe weight loss and death^16^. However, amino acid mutation at the key site of neuraminidase (NA) may lead to reduced susceptibility of influenza virus to NAIs and thus weaken the therapeutic effect^17^. There are 19 highly conserved residues in the NA active site of all influenza A and B viruses. These include eight catalytic residues (R118, D151, R152, R224, E276, R292, R371, and Y406) that directly contact the sialic acid (SA) and 11 framework residues (E119, R156, W178, S179, D198, I222, E227, H274, E277, N294, and E425) that support the enzymatic binding pocket^18,19^. When the conserved amino acids in the NA active site of influenza virus mutated, the susceptibility to NAIs may be reduced. NA R292K substitution could lead to resistance among multiple NA subtypes of influenza virus, but other resistance mutation sites may be subtype-specific^20,21^. Recombinant N9 proteins bearing substitutions showed that R292K, H274Y conferred high increase in oseltamivir half-maximal inhibitory concentration (IC_50_), and E119D conferred high zanamivir IC_50_. Additionally, R152Kof A (H7N9) virus conferred reduced inhibition by laninamivir^22^.

Our previous research found that the rate of strains isolated from human with reduced susceptibility to NAIs in HPAI H7N9 virus was high^23^. HPAI H7N9 viruses with reduced susceptibility to neuraminidase inhibitors (NA R292K, E119V or H274Y) showed good replication capacity in mammalian cells^24^. Interestingly, NA 119V or D substitutions in HPAI H7N9 showed reduced susceptibility to oseltamivir and zanamivir respectively^24^. Like E119 amino acids, I222 amino acids are also located in the frame region of NA, and it has been reported that I222 substitutions combined with other frame region resistance sites have been detected in seasonal influenza cases^25,26^. In this study, we want to test if the two framework mutations (E119V and I222L) of NA could coexist in HPAI H7N9 virus, and study its effect on viral fitness and drug resistance effect in mammalian cells and mice.

## Results

### Generation of recombinant viruses

In this study, A/Guangdong/17SF006/2017 (H7N9) was used as a backbone. The NAIs-sensitive HPAI H7N9 virus constructed by reverse genetics containing NA 119E, 222I, was called rg006NA. The HPAI H7N9 reassortment viruses bearing substitutions were called rg006NA119V, rg006NA222L and rg006NA222L-119V according to their mutations.

The H7N9/PR8 reassortment virus and its NA mutants were called rg006NA/PR8, rg006NA119V/PR8, rg006NA222L/PR8 and rg006NA222L-119V/PR8 respectively. The sequencing of H7N9/PR8 recombinant viruses showed that 339–342KRTA polybasic amino acids in HA had been knocked out correctly, and all the six internal genes were from PR8. The four recombinant H7N9/PR8 viruses of rg006NA/PR8, rg006NA119V/PR8, rg006NA222L/PR8 and rg006NA222L-119V/PR8 were cultured at 35 °C for 48 h at a dilution of 10^–1^–10^–6^, and none were lethal to 9–11 day embryonated chicken eggs. The results of trypsin dependence test showed that all the four H7N9/PR8 recombinant viruses could not replicate without TPCK-trypsin, suggesting that the virus was dependent on TPCK-trypsin. The results showed that the recombinant H7N9/PR8 virus was attenuated successfully.

The recombinant viruses used in this study were confirmed by sequencing and listed in Supplementary Table S1.

### Assessment of susceptibility to neuraminidase inhibitors

The susceptibility of HPAI H7N9 reassortment viruses to NAIs was assessed (Table 1). The substitution of E119V in the NA protein induced a mean 88.03-fold and 6.12-fold increase in the IC_50_ of oseltamivir (mean IC_50_ (nM) ± SD, 141.73 ± 10.52) and zanamivir (mean IC_50_ (nM) ± SD, 11.81 ± 0.32) respectively. The IC_50_ fold change of oseltamivir or zanamivir to rg006NA222L were 4.50 and 2.46 times respectively, which did not meet the phenotype resistance standard. However, when I222L and E119V substitutions coexist in the NA protein of HPAI H7N9 virus, the IC_50_ fold change of oseltamivir to rg006NA222L-119V is 306.34 (mean IC_50_ (nM) ± SD, 493.20 ± 29.10) times than that of its susceptible strain, and 3.5 times than that of the E119V single point mutant virus. The result showed that NA I222L-E119V substitutions had synergistic resistance effect to oseltamivir for HPAI H7N9 virus. On the other hand, rg006NA222L-119V induced a mean 7.53-fold increase in the IC_50_ of zanamivir, which is similar to the value of E119V single point mutant virus. Besides, the fold change of IC_50_ value of all the three mutant viruses to peramivir and laninamivir was less than 10, suggesting normal inhibition.

**Table 1:** Susceptibility of mutant recombinant highly pathogenic H7N9 avian influenza virus to neuraminidase inhibitors.

Viruses	Oseltamivir		Zanamivir		Peramivir		Laninamivir	
	Mean IC50^a^ (nM) ± SD	Fold change^b^	Mean IC50^a^ (nM) ± SD	Fold change^b^	Mean IC50^a^ (nM) ± SD	Fold change^b^	Mean IC50^a^ (nM) ± SD	Fold change^b^
rg006NA(222I-119E)	1.61 ± 0.04	1.00	1.93 ± 0.09	1.00	0.28 ± 0.02	1.00	0.72 ± 0.04	1.00
rg006NA119V	141.73 ± 10.52	88.03^c^	11.81 ± 0.32	6.12	0.48 ± 0.01	1.71	3.26 ± 0.02	4.53
rg006NA222L	7.25 ± 0.79	4.50	4.75 ± 0.38	2.46	0.28 ± 0.02	1.00	1.23 ± 0.06	1.71
rg006NA222L-119V	493.20 ± 29.10	306.34^c^	14.53 ± 1.25	7.53	0.52 ± 0.01	1.86	3.14 ± 0.04	4.36

^a^IC_50_, half-maximal inhibitory concentration. The IC_50_ denotes the concentration of a NA inhibitor that reduces the NA activity by 50% relative to NA activity without the inhibitor.

^b^Fold change relative to the mean IC_50_ of the wild-type NA protein. Fold-change values of each NA were interpreted using criteria established by the World Health Organization Influenza Antiviral Working Group.

^c^Fold change >10 (including reduced/highly reduced inhibition).

### Growth characteristics of mutant viruses in MDCK cells

**Figure 1:**
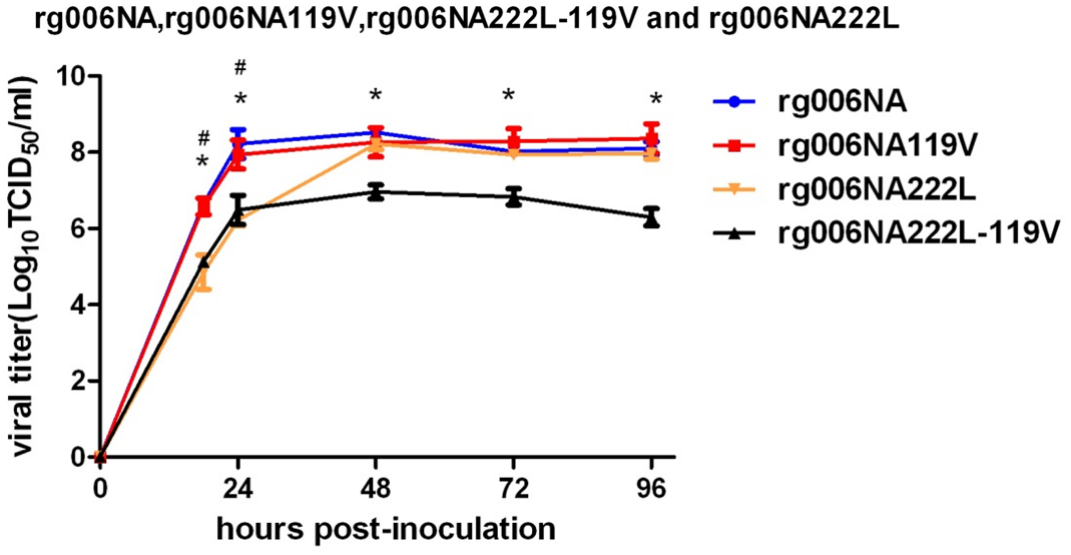
Replication kinetics of mutant recombinant highly pathogenic H7N9 avian influenza viruses in MDCK cells. Cells were inoculated with the recombinant viruses at 37 °C, and culture supernatants were harvested at 0, 18, 24, 48, 72 and 96 h post-inoculation (hpi). Virus titers were determined on MDCK cells. Growth curve of rg006NA in MDCK cells was in blue, round; rg006NA119V virus was in red, square; rg006NA222L virus was in orange, triangle; rg006NA222L-119V virus was in black, triangle. Each data point represents the mean log_10_ ± SD TCID_50_/ml from at least two independent tests. ^#^P < 0.05. *P < 0.05.

To test the effect of the NAIs resistance mutations on the growth characteristics of the HPAI H7N9 virus, we determined infectious titers in MDCK cells. MDCK cells were infected with rg006NA, rg006NA119V, rg006NA222L and rg006NA222L-119V virus by 0.001 MOI. At time points 0, 18, 24, 48, 72 and 96hpi, viral titers were determined on MDCK cells. Rg006NA and rg006NA119V virus showed similar replication level in MDCK cells at different time points (P > 0.05) (Fig. 1). The replication level of rg006NA222L was lower than that of rg006NA in 18 and 24hpi (^#^*P* < 0.05), but they showed similar replication level after 48hpi in MDCK cells. Replication peak value of rg006NA222L-119V reached more than 6log_10_ TCID_50_/ml in 48 h although its replication level was lower than that of rg006NA(*P < 0.05). The results showed that rg006NA222L-119V, the two sites mutant virus with synergistic drug resistance effect to NAIs, had good replication ability in mammalian cells though lower than that of the sensitive strain.

### Pathogenicity in mice

**Figure 2:**
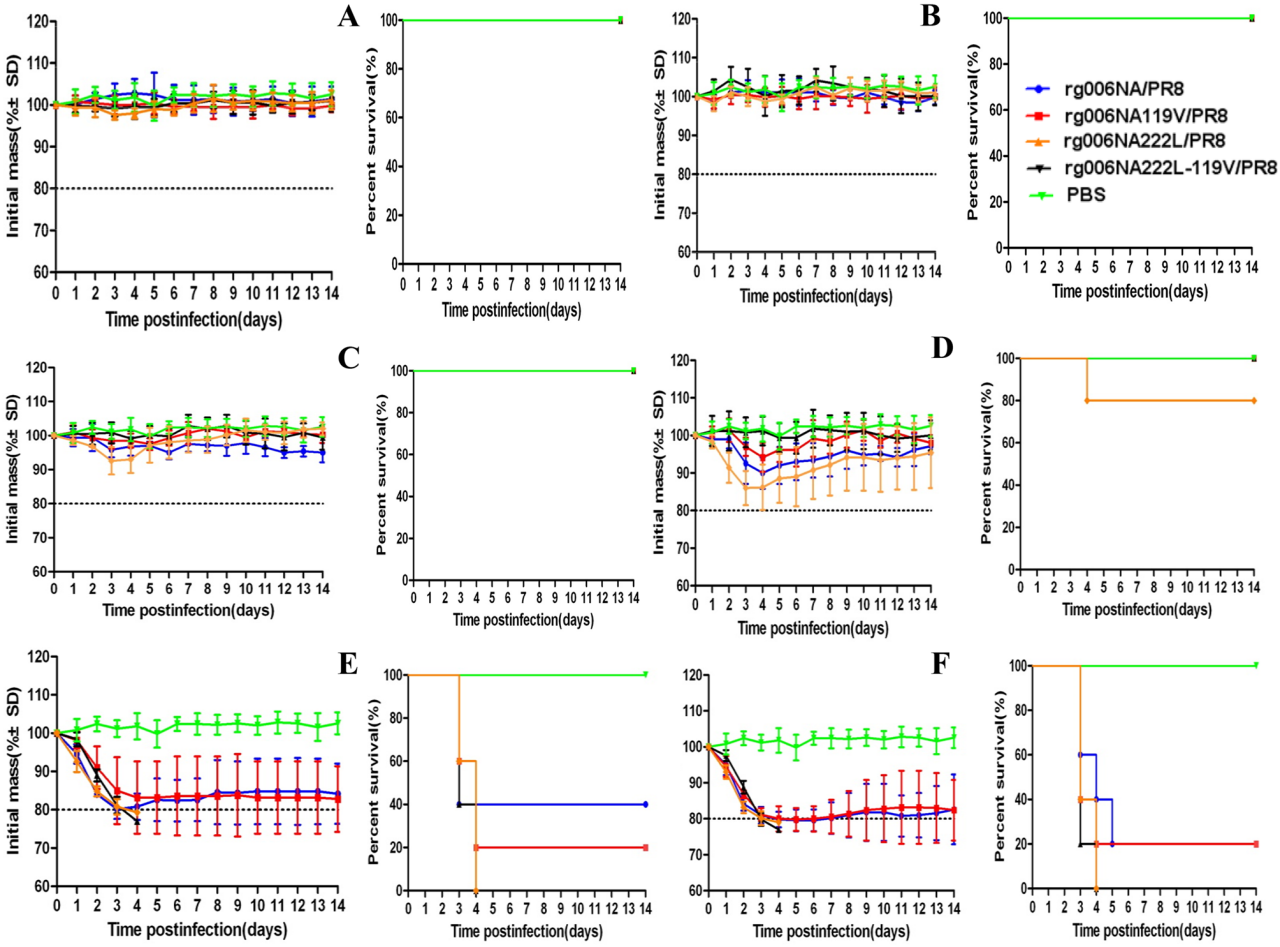
Body weight curve and survival condition of mice infected with different doses of H7N9/PR8 recombinant viruses. Body weight change and survival condition of C57BL/6 mice (n = 5/group) inoculated intranasal with 10^1^–10^6^ TCID_50_ recombinant H7N9/PR8 viruses. Control group: PBS. (**A**) 10^1^ TCID_50_; (**B**) 10^2^ TCID_50_; (**C**) 10^3^ TCID_50_; (**D**) 10^4^ TCID_50_; (**E**) 10^5^ TCID_50_; (**F**) 10^6^ TCID_50_.

The body weight curve and survival condition of 8-week-old female C57 mice infected with 10^1^–10^6^ TCID_50_ recombinant H7N9/PR8 viruses are shown in Fig. 2.When infection at a lower dose (≤ 10^4^ TCID_50_), all mice infected with these four strains survived except one mouse in the 10^4^ TCID_50_ rg006NA222L/PR8 group (Fig. 2A–D). Rg006NA119V-222L/PR8 viruses showed no obvious weight loss at lower dose (≤ 10^4^ TCID_50_) (Fig. 2A–D). However, When the infection dose reached10^5^–10^6^ TCID_50_, all the four viruses could lead to significant weight loss and fatality in mice (Fig. 2E,F). There was no significant difference in the survival day of mice among the four viruses from 10^1^ TCID_50_–10^6^ TCID_50_ (*P* > 0.05). There was no significant difference of hazard ratio between the mutant virus (rg006-NA119V/rg006NA222L/PR8/rg006NA119V-222L/PR8) and rg006NA/PR8 (P > 0.05).The survival ratio of rg006NA222L-119V/PR8 virus at 10^5^–10^6^ TCID_50_ was 0% (the same as rg006NA222L/PR8), which was even lower than that of rg006NA (10^5^ TCID_50_: 40%; 10^6^ TCID_50_: 20%) and rg006NA119V (10^5^ TCID_50_: 20%; 10^6^ TCID_50_: 20%) (Table 2).

**Table 2:** Pathogenicity of the reassortment H7N9/PR8 influenza viruses in a C57 mouse model.

Virus dose (TCID_50_)	rg006NA			rg006NA119V				rg006NA222L				rg006NA222L119V			
	No. of mice survived/total no.	Survival rate (%)	Mean survival day	No. of mice survived/total no.	Survival rate (%)	Mean survival day	Hazard ratio^a^	No. of mice survived/total no.	Survival rate (%)	Mean survival day	Hazard ratio^b^	No. of mice survived/total No	Survival rate (%)	Mean survival day	Hazard ratio^c^
10^1^	5/5	100	14 ± 0	5/5	100	14 ± 0	1	5/5	100	14 ± 0	1	5/5	100	14 ± 0	1
10^2^	5/5	100	14 ± 0	5/5	100	14 ± 0	1	5/5	100	14 ± 0	1	5/5	100	14 ± 0	1
10^3^	5/5	100	14 ± 0	5/5	100	14 ± 0	1	5/5	100	14 ± 0	1	5/5	100	14 ± 0	1
10^4^	5/5	100	14 ± 0	5/5	100	14 ± 0	1	5/5	80	12 ± 4	65.29, 95% CI 0–6.28e8	4/5	100	14 ± 0	1
10^5^	2/5	40	7 ± 5	1/5	20	6 ± 4	1.19, 95% CI 0.27–5.35	0/5	0	4 ± 0	1.44, 95% CI 0.34–6.06	0/5	0	3 ± 0	1.67, 95% CI 0.40–6.97
10^6^	1/5	20	4 ± 1	1/5	20	5 ± 4	2.24, 95% CI 0.51–9.75	0/5	0	3 ± 0	1.95, 95% CI 0.46–8.21	0/5	0	3 ± 0	2.24, 95% CI 0.51–9.75

^a^rg006-NA119V vs rg006-NA, Cox regression analysis by SPSS software.

^b^rg006-NA222L vs rg006-NA, Cox regression analysis by SPSS software.

^c^rg006-NA222L-119V vs rg006-NA, Cox regression analysis by SPSS software.

The mouse lethal dose of 50% animals (MLD_50_) of rg006NA/PR8, rg006NA119V/PR8, rg006NA222L/PR8 and rg006NA222L-119V/PR8 virus to C57 mice was 10^5^ TCID_50_, 10^4.75^ TCID_50_, 10^4.38^ TCID_50_ and 10^4.5^ TCID_50_, respectively.

**Figure 3:**
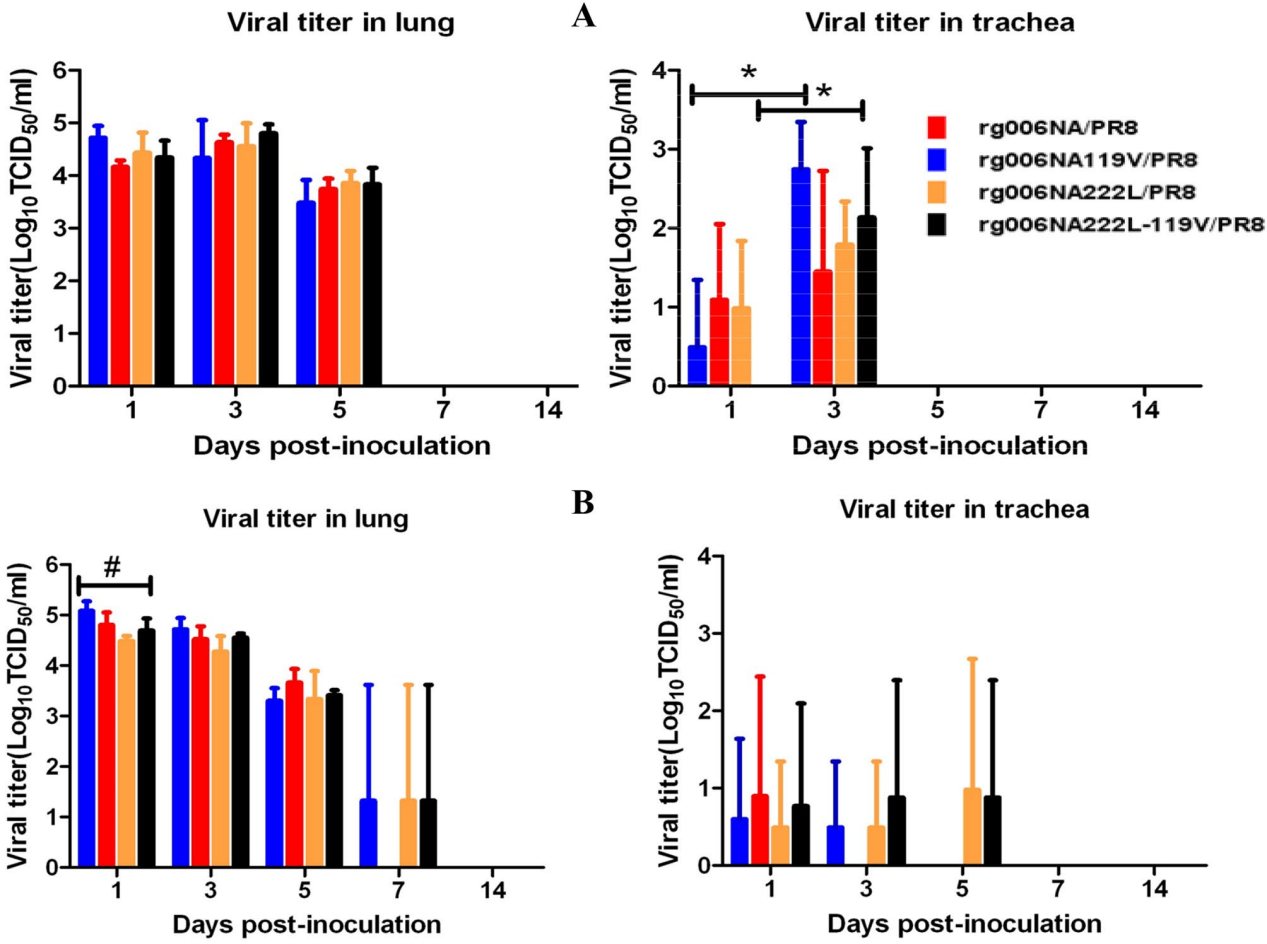
Replication of H7N9/PR8 recombinant viruses in the respiratory tract of mice. C57BL/6 mice (n = 3/time-point) were inoculated intranasal with 10^4^ TCID_50_ (**A**) or 10^5^ TCID_50_ (**B**) of the H7N9/PR8 recombinant viruses. Mice were euthanized at 1, 3, 5, 7 and 14 dpi. Viral titers in the lung (lu) or tracts (tr) were determined on MDCK cells. *P < 0.05. ^#^P < 0.05.

The viral load of rg006NA/PR8, rg006NA119V/PR8, rg006NA222L/PR8 and rg006NA222L-119V/PR8 in the respiratory tract of mice were shown in Fig. 3. ANOVA test was used to analyze the difference of viral load of the 4 strains in different tissues (lung and trachea) at different time points (1, 3, 5, 7, 14 dpi) and the result showed that there was no significant difference among the 4 viruses (*P* > 0.05) except that in lung tissue 1dpi (^#^*P* < 0.05) of the 10^5^ TCID_50_ group (Fig. 3B). When administered with 10^4^ TCID_50_ of the 4 viruses, the viral load in trachea of the mice infected with rg006NA/PR8 and rg006NA222L-119V/PR8 was higher at 3dpi than that at 1dpi (*P < 0.05), which indicated viral replication, and on 5dpi, no viral load was detected in trachea. When administered with 10^5^ TCID_50_, the viral load in trachea of the mice decreased from 1 to 3 dpi, and on 5dpi it could only be detected in one mouse in the rg006NA222L-119V/PR8 and the rg006NA222L/PR8 group respectively. And on 7 dpi, no viral load was detected in trachea of all the mice. When administered with 10^4^ TCID_50_ or 10^5^ TCID_50_ of the 4 viruses, the viral load in lungs of mice was high at 1 dpi and 3 dpi, and then decreased from 5 dpi. On 7 dpi, viral load in lung could only been detected in one mouse infected with rg006NA/PR8, rg006NA222L/PR8 or rg006NA222L-119V/PR8 virus in the 10^5^ TCID_50_ dose group respectively. On 14 dpi, no virus load was detected in lungs of all the mice. In general, the viral load in the respiratory tract of mice infected with rg006NA/PR8, rg006NA119V/PR8, rg006NA222L/PR8 and rg006NA222L-119V/PR8 virus bore out similar trend.

Through the detection of HA antibody in mouse serum (serum antibody titer shown in Supplementary Table S2), we calculated that the mouse infective dose of 50% animals (MID_50_) of rg006NA/PR8, rg006NA119V/PR8, rg006NA222L/PR8 and rg006NA222L-119V/PR8 was 0.83 TCID_50_, 1 TCID_50_, 0.83 TCID_50_ and 1.38 TCID_50_ respectively. The results showed that the infectivity of the four viruses to mice was similar and all of them can infect mice at a low dose.

**Figure 4:**
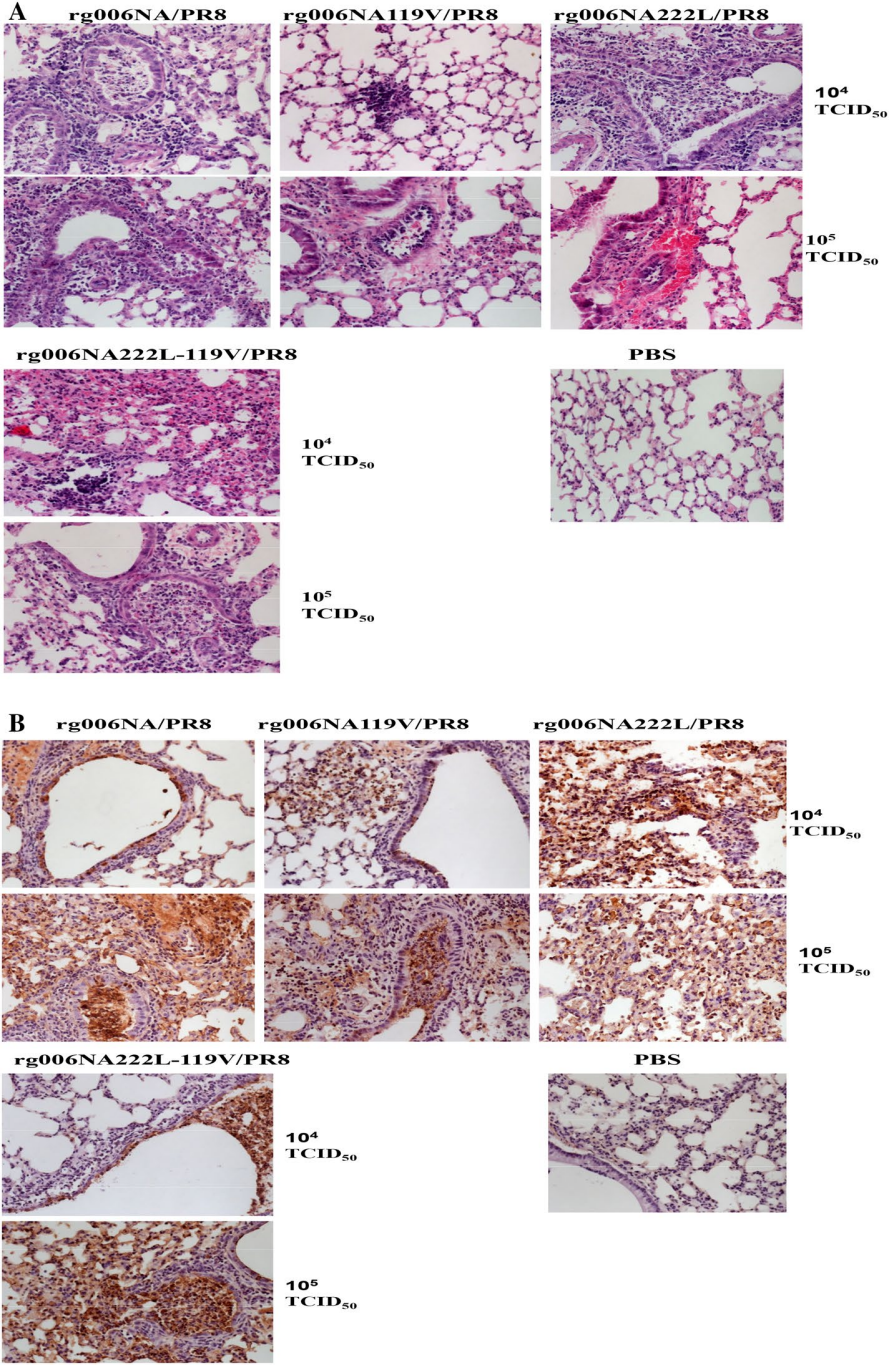
Pathological changes of lung tissue in mice infected with H7N9/PR8 recombinant viruses. The lung tissues of mice infected with 10^4^–10^5^ TCID_50_H7N9/PR8 recombinant viruses were stained by hematoxylin and eosin ((**A**) HE × 20) and NP antigen ((**B**) IHC × 20) 3 days after infection. Control group: PBS.

Lungs of mice infected with 10^4^ and 10^5^ TCID_50_ viruses on 3dpi were studied for pathology. In Fig. 4A, the lung tissue of PBS control group of mice showed normal shape without obvious change. The lung tissues infected with the 4 viruses showed inflammatory cell infiltration, alveolar injury and other pathological changes. At 10^4^ TCID_50_, there were some inflammatory cells in lung tissues of mice infected with the four viruses. At 10^5^ TCID_50_, all the lung tissues infected with the 4 viruses exhibited much inflammatory infiltration and extensive pneumonia. Immunohistochemistry results shown in Fig. 4B, there is no NP antigen distribution in the lung tissue of PBS control group mice. After 10^4^ TCID_50_ doses of infection, the lung tissues infected with the 4 viruses were stained with scattered NP antigens. A large number of NP antigens were found in lung tissues infected with the 4 viruses when infected with 10^5^ TCID_50_, which were widely distributed.

In conclusion, rg006NA/PR8, rg006NA119V/PR8, rg006NA222L/PR8 and rg006NA222L-119 V/PR8 viruses had similar pathogenicity to mice.

### Synergistic resistance to oseltamivir in mice

**Figure 5:**
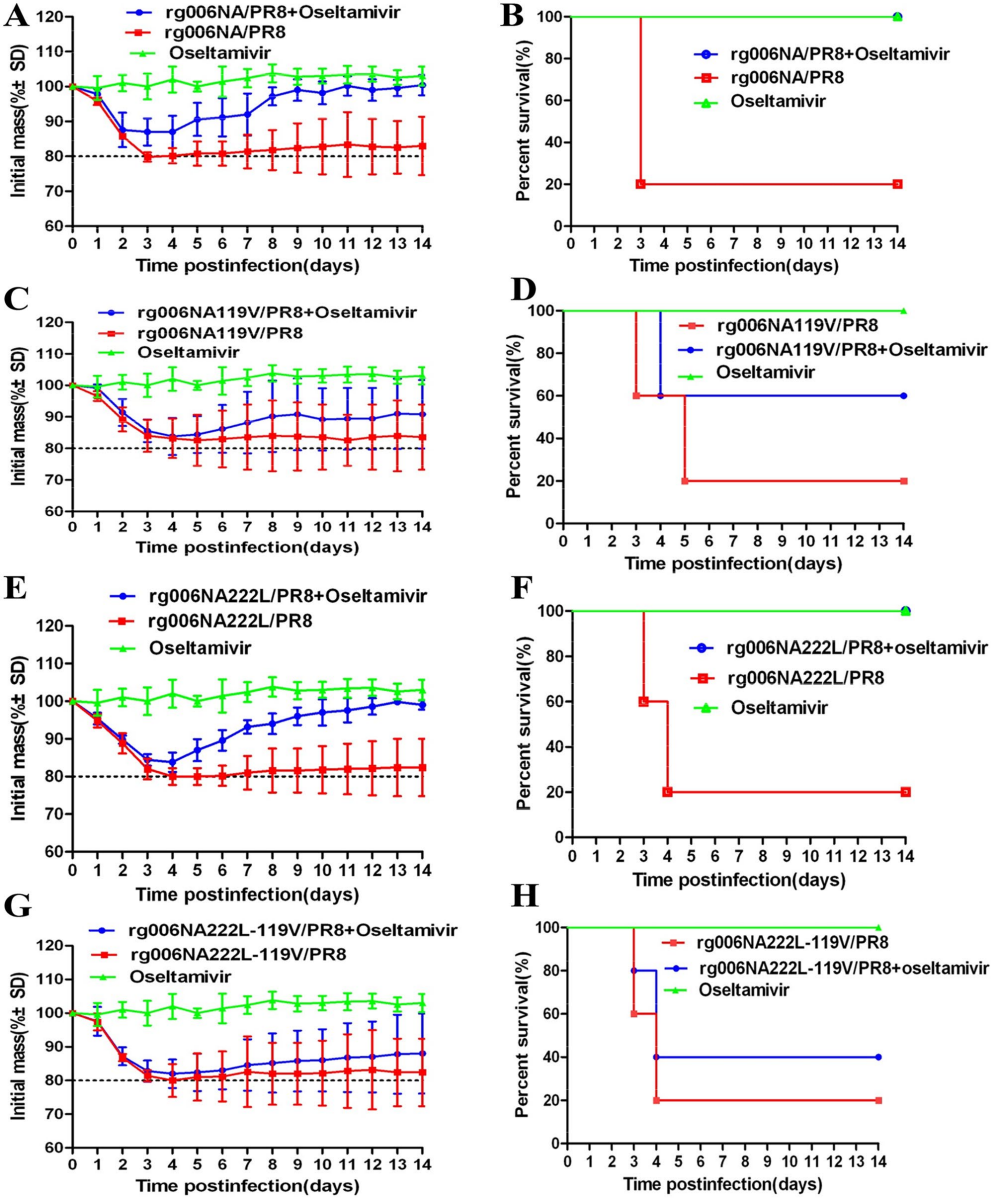
Body weight curve and survival condition of mice infected with H7N9/PR8 recombinant viruses treated with oseltamivir phosphate. The C57 mice were infected with 2MLD_50_ rg006NA/PR8, rg006NA119V/PR8, rg006NA222L-119V/PR8 and rg006NA222L/PR8 (n = 5/group). The mice in the drug treatment group were given 20 mg/kg/day oseltamivir phosphate by gavage from 0 to 4 dpi, and the mice in the non-drug group were given PBS by gavage from 0 to 4 dpi. On the day of infection, the mice in the control group were given PBS, and then the mice were given 20 mg/kg/day oseltamivir phosphate by gavage. (**A,C,E,G**) weight curve of mice; (**B,D,F,H**) survival condition of mice. (**A,B**) rg006NA/PR8; (**C,D**) rg006NA119V/PR8; (**E,F**) rg006NA222L/PR8; (**G,H**) rg006NA222L-119V/PR8.

We infected mice with 2MLD_50_ of the 4 viruses. As shown in Fig. 5, administrated with oseltamivir 20 mg/kg/day by gavage could prevent rg006NA/PR8 or rg006NA222L/PR8 infected mice from much body weight loss and fatality (Fig. 5A,B,E,F). The survival day of rg006NA/PR8 and rg006NA222L/PR8 infected mice given oseltamivir treatment was longer than their non-treatment counterparts (*P < 0.05). There was no statistical difference between the survival day of rg006NA119V/PR8 and rg006NA222L-119V/PR8 infected mice in oseltamivir or non-treatment group (*P* > 0.05). There was no significant difference of hazard ratio between the oseltamivir-treated and non-treated group of all the four viruses (P > 0.05) (Table3). The rg006NA119V/PR8 infected mice by oseltamivir administration had a slight alleviation of body weight loss and fatality compared with the non-medication group (Fig. 5C,D). However, the rg006NA222L-119V/PR8 infected mice got the resemble body weight loss no matter whether or not to give oseltamivir treatment (Fig. 5G). The mortality rate of rg006NA222L-119 V/PR8 mice with or without drug administration was 60% and 80% respectively (Fig. 5H). The results showed that oseltamivir had poor therapeutic effect on rg006NA119V/PR8 and even poorer therapeutic effect on rg006NA222L-119V/PR8 virus, which could not effectively protect mice from weight loss and death.

**Table 3:** Survival of mice in drug or non-drug groups.

	rg006NA/PR8		rg006NA119V/PR8		rg006NA222L/PR8		rg006NA222L-119V/PR8	
	PBS	Oseltamivir	PBS	Oseltamivir	PBS	Oseltamivir	PBS	Oseltamivir
No. of mice survived	1/5	5/5	1/5	3/5	1/5	5/5	1/5	2/5
Survival rate	20%	100%	20%	60%	20%	100%	20%	40%
Mean survival days	5 ± 4*	14 ± 0*	6 ± 4	10 ± 5	6 ± 4*	14 ± 0*	6 ± 4	8 ± 5
Hazard ratio	0.02, 95% CI 0–47.51		0.42, 95%CI 0.08–2.31		0.01, 95% CI 0–40.07		0.64, 95% CI 0.14–2.85	

*P < 0.05.

**Figure 6:**
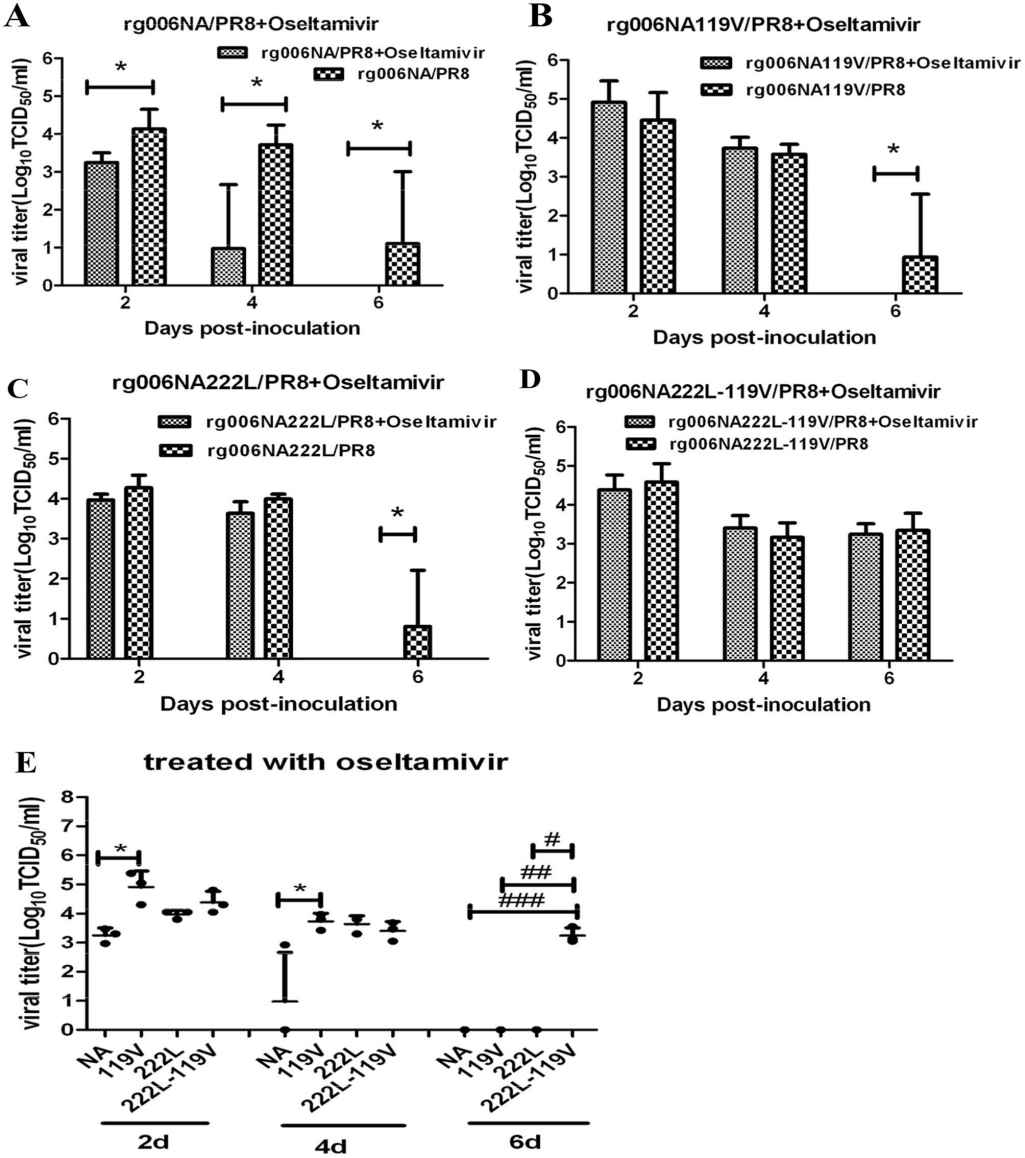
Viral load of lung tissue of mice infected with H7N9/PR8 recombinant viruses treated with oseltamivir phosphate. The lung tissues of C57 mice infected with 2MLD_50_ H7N9/PR8 recombinant viruses were titrated 2, 4 and 6 dpi (n = 3/time-point). Mice in the drug treatment group: 20 mg/kg/day oseltamivir phosphate by gavage from 0 to 4dpi; Mice in the non-drug group: PBS by gavage from 0 to 4 dpi. (**A–D**) Comparison of mice infected with H7N9/PR8 recombinant viruses in the drug treatment group or non-drug group. (**E**) Comparison of mice infected with rg006NA/PR8 (NA), rg006NA119V/PR8 (119V) and rg006NA222L-119V/PR8 (222L-119 V) virus after oseltamivir administration. *P < 0.05. ^#^*P* < 0.05. ^##^*P* < 0.05. ^###^*P* < 0.05.

The viral load of rg006NA/PR8, rg006NA119V/PR8, rg006NA222L/PR8 and rg006NA222L-119V/PR8 in lungs of mice with oseltamivir administration are shown in Fig. 6. The rg006NA/PR8 infected mice had significantly lower viral load in lung by oseltamivir administrated than the non-medication group (2, 4 and 6 dpi, *P* < 0.05, Fig. 6A). There is no difference of viral load in lung between the treated and the non-treated mice which infected with rg006NA119V/PR8 or rg006NA222L/PR8 until 6 dpi (*P* < 0.05, Fig. 6B, C). Mice infected with rg006NA222L-119V/PR8 virus had similar viral load in lung at 2, 4 and 6 dpi no matter whether given oseltamivir or not (Fig. 6D). At 2 and 4 days of oseltamivir administration, the viral load of rg006NA119V/PR8 infected mice was higher than the rg006NA/PR8 group (*P* < 0.05, Fig. 6E). At 6 days of oseltamivir administration, the viral load of rg006NA222L-119V/PR8 infected mice was higher than the rg006NA/PR8, rg006NA119V/PR8 or rg006NA222L/PR8 group (*P* < 0.05, Fig. 6E).

**Figure 7:**
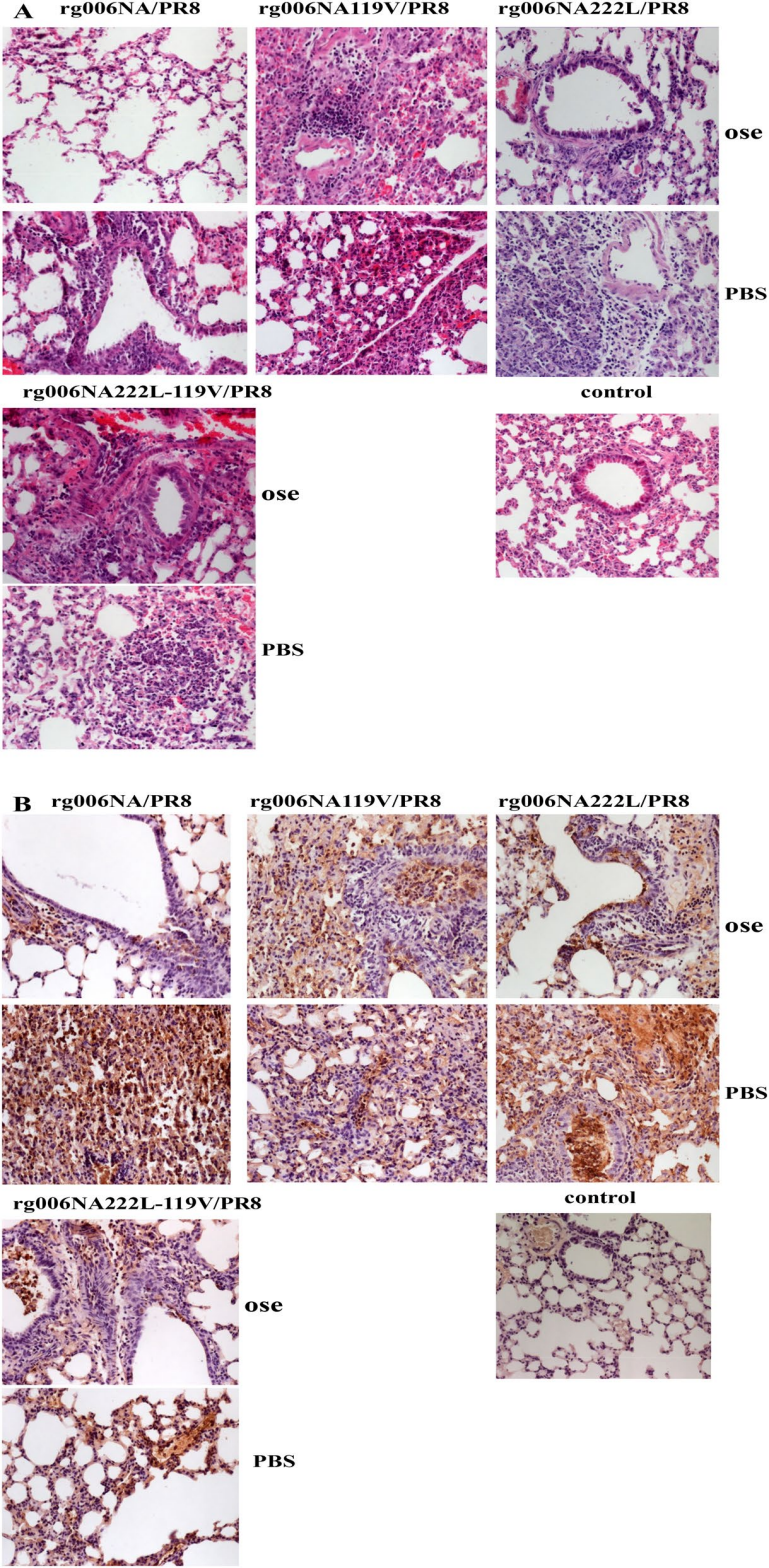
Pathological changes of lung tissue of mice infected with H7N9/PR8 recombinant viruses treated with oseltamivir phosphate. The lung tissues of mice infected with H7N9/PR8 recombinant viruses were stained by hematoxylin and eosin ((**A**) HE × 20) and NP antigen ((**B**) IHC × 20) 4 days after infection. Ose (oseltamivir treatment group): 2MLD_50_ rg006NA/PR8, 20 mg/kg/day, rg006NA119V/PR8, rg006NA222L/PR8 or rg006NA222L-119V/PR8 infection, oseltamivir phosphate by gavage from 0 to 4 dpi; PBS(control group): 2MLD_50_ rg006NA/PR8, 20 mg/kg/day, rg006NA119V/PR8, rg006NA222L-119V/PR8 or rg006NA222L/PR8 infection, PBS by gavage from 0 to 4 dpi. Control group, PBS, 20 mg/kg/day oseltamivir phosphate by gavage from 0 to 4dpi.

Four days after infection, lungs of rg006NA/PR8, rg006NA119V/PR8, rg006NA222L/PR8 and rg006NA222L-119V/PR8 infected mice with oseltamivir administration were taken for pathological observation. By HE staining (Fig. 7A), without medication, mice infected with the four viruses all developed diffuse pneumonia with a large number of inflammatory cell infiltration. Under the condition of oseltamivir, only a small number of inflammatory cells were found in the lung of rg006NA/PR8 infected mice which was obviously milder inflammatory than the non-drug group. As to rg006NA222L/PR8 virus, after drug treatment, the pathological changes of lung in mice were significantly alleviated. However, there was no significant difference in lung pathological damage between the oseltamivir-treated and non-treated mice which infected with rg006NA119V/PR8 and rg006NA222L-119V/PR8 virus. As a PBS control group, oseltamivir by gavage made no obvious pathological changes in the lung tissue of mice.

The distribution of NP antigen was consistent with the above result, that the use of oseltamivir reduced the lung antigens in mice infected with rg006NA/PR8 and rg006NA222L/PR8 (Fig. 7B), but did not significantly reduce the antigens in mice infected with rg006NA119V/PR8 and rg006NA222L-119V/PR8. As a PBS control group, there is no antigen in the lung tissue of mice.

In all, E119V substitution in NA made the H7N9/PR8 reassortment virus resistant to oseltamivir in mice. What’s more, NA substitutions of I222L and E119V had synergistic resistance effect on the H7N9/PR8 reassortment virus to oseltamivir in mice.

## Discussion

Generally, when influenza viruses possess drug resistant mutations, their replication level in cells or their pathogenicity to animals may be reduced to varying degrees, especially when mutations occur in catalytic residues that directly contact the sialic acid (SA)^19,27^. After a single site mutation of R292K, the enzyme activity of HPAI H7N9 virus decreased significantly and its replication ability decreased to some extent^19^. According to the substitution with reduced susceptibility to NAIs detected in HPAI H7N9 cases^24^, we used reverse genetic technology to develop double sites mutant HPAI H7N9 viruses carrying I222L-119V, H274Y-R292K and E119V-R292K, but only the enzyme activity of rg006NA222L-119V virus was enough to perform the NA inhibition test.

Seasonal influenza viruses bearing two mutations in the frame region of NA had been report before. A H3N2 virus possessing the mutations E119V-I222V, isolated from human cases and induced a synergistic effect on oseltamivir resistance test in vitro^26^. Besides, prophylactic treatment with oseltamivir had failed in two H1N1 with H274Y-I222M mutations infected patients^25^. These results show that the two site mutations in the framework region can coexist in influenza viruses, and some of them have synergistic drug resistance effect. However, these studies are limited to clinical discovery or cell level studies of seasonal influenza viruses, and there are few studies of influenza virus with double resistance sites in a mouse model.

The E119 residue is conserved among all influenza A viruses and forms a hydrogen bond with the hydroxyl group on the C-4 of sialic acid^28^ and with the amino group on C-4 of oseltamivir^29^. E119A/D/G/I/V substitutions have been examined conferring resistance to NAIs in influenza viruses of various subtypes^22,24,30–34^. And in this study, E119V substitution in HPAI H7N9 virus induced a mean 88.03-fold increase in the IC_50_ of oseltamivir. The I222 residue is a conserved residue in NA of influenza viruses and I222V/M/L/R/T substitutions had been reported with the N1, N2, and B backgrounds conferring moderate resistance to oseltamivir^31,33–36^. However, NA I222L substitution of HPAI H7N9 did not lead to drug resistance to oseltamivir, zanamivir, peramivir and laninamivir according to WHO-AVWG criteria. In this study, the two mutations of E119V-I222L were chosen to study the synergistic drug resistance of HPAI H7N9 virus in mice. The IC_50_ fold change of oseltamivir to rg006NA222L-119V is 306.34 times than that of its susceptible strain, and 3.5 times than that of the E119V single point mutant virus, which showed synergistic resistance effect. A crystal structure model of the NA monomer of the A/Anhui/1/2013 H7N9 virus (PDB ID: 4MWJ) was used to display and label the two mutations (E119V and I222L) in H7N9 virus (seen in Supplementary Fig. S1). Both 119V and 222L which located around the catalytic sites of NA were framework sites that interact with catalytic residues to stabilize the active site.

NAIs development is structure-based drug design^37^. As a surface glycoprotein with enzyme activity, NA is an ideal target for the design of competitive inhibitors^38^. NAIs bind to the NA active site with a higher affinity than the natural SA and prevent the release of new virions from infected cells^39^. Oseltamivir (oseltamivir phosphate) is taken orally and then converted to its active form (oseltamivir carboxylate) to disseminate systemically. The recommended oral dose for adolescents and adults is 75 mg oseltamivir twice daily for 5 days^40^. Excessive concentration of oseltamivir is not conducive to antiviral treatment^16,41–43^. The concentration of oseltamivir selected in this study is 20 mg/kg/day, which is feasible and effective in this study. Consistent with the result of NA inhibition test in vitro, E119V-I222L in N9 from HPAI H7N9 virus have synergistic drug resistance effect to oseltamivir in mice (Figs. 5, 6). When infected at 10^4^ TCID_50_, the pathogenicity of the rg006NA222L-119V/PR8 virus to mice is slightly lower (less weight loss) compared with rg006NA119V/PR8, rg006NA222L/PR8 and their sensitive strain (Fig. 2D), which is basically the same as that on MDCK cells (Fig. 1). Significantly, when the infection dose reached10^5^–10^6^ TCID_50_, rg006NA222L-119V/PR8 (and rg006NA222L/PR8) virus even lead to more weight loss and fatality rate than that of rg006NA/PR8 and rg006NA119V/PR8 in mice (Fig. 2E,F). It had been reported that NA-I222K/R substitution of A (H7N9) made the virus more virulent (weight loss and survival rate) in mice but not in MDCK cells^44^, which was consistent with our study. It was suggested that NA I222L substitution made rg006NA222L-119V/PR8 virus more virulent in mice. Generally speaking, there was no significant difference in the survival day (10^1^ TCID_50_–10^6^ TCID_50_), lung/trachea virus load and lung pathological changes among the four virus infection mice groups. The N9 E119V-I222L virus which had synergistic drug resistance effect did not seriously damage of its fitness in mice.

In terms of bio-safety and practical conditions, the recombinant viruses used in the mouse experiment were attenuated. Compared with their corresponding HPAI H7N9 viruses, the replication ability on MDCK cells of the attenuated H7N9/PR8 recombinant viruses decreased to some extent (data not shown). The pathogenicity of the H7N9/PR8 viruses was weaker than their corresponding HPAI H7N9 viruses in mice. This is one of the limitations of this study. However, the H7N9/PR8 recombinant viruses developed in this study could replicate in respiratory tract of C57 mice and lead to pneumonia, weight loss and even death, indicating the C57 mice infected with H7N9/PR8 recombinant viruses with reduced susceptibility to NAIs were feasible model for the study on the mechanism of HPAI H7N9 NAIs resistance and the preliminary selection of alternative drugs.

In summary, although NA I222L did not lead HPAI H7N9 virus to drug resistance itself, it could increase the resistance of HPAI H7N9 virus with NA E119V without severely impaired fitness in vitro. Consistent with results in vitro, substitutions of I222L and E119V in neuraminidase from HPAI H7N9 influenza virus exhibited a synergistic resistance effect to oseltamivir without significantly reduced pathogenicity in mice. Due to the good fitness of drug resistant strains of HPAI H7N9 virus, it is necessary to strengthen drug resistance surveillance, as well as research and application of new drugs.

## Materials and methods

All experiments including any relevant details were approved by designated institution and/or licensing committee, and all experiments were performed in accordance with the relevant guidelines.

### Generation of recombinant viruses

The generation of HPAI H7N9 recombinant viruses was described in Tang et al.^24^. In short, HPAI H7N9 A/Guangdong/17SF006/2017 (abbreviated as 006) isolated from a human case was used as backbone virus. All eight gene segments of 006 strain were artificially synthesized (Sangon Biotech) and cloned into the pHW2000 vector. The NAIs sensitive HPAI H7N9 reassortant virus encoded 222I and 119E in the NA protein. A single-point mutation was introduced to convert 119E to 119V or 222I to 222L in NA. Then, in the 119V background, single-point mutations I222L in the NA gene were carried out. E119V or I222L substitutions were generated with the following primers:

- NA E119V Forward, gtcgcatgaaacatagggtactcttgtgactaaaacatc,
- NA E119V Reverse, gatgttttagtcacaagagtaccctatgtttcatgcgac,
- NA I222L Forward, acacatgggcccgaaacttactaagaacacaggaa,
- NA I222L Reverse, ttcctgtgttcttagtaagtttcgggcccatgtgt.

Mutations were introduced into the NA plasmid using the QuikChange Site-Directed Mutagenesis Kit (Stratagene). HPAI H7N9 reassortment viruses were used to examine the effect of NA amino acid substitutions on NAIs susceptibility and viral replication efficiency in vitro.

Considering the bio-safety and actual conditions, attenuated H7N9 reassortment viruses were prepared to evaluate the pathogenicity and drug resistance in mice. The internal six genes of the attenuated virus were from A/PR/8/34 (H1N1). The HA gene was from 006strain after deletion of its highly pathogenic molecular markers (KRTA in 339–342 residue). The recombinant H7N9/PR8 attenuated virus was prepared by transfection of the above seven plasmids and 006 NA plasmids. HA 339–342KRTA deletion was generated with the following primers:

- HAdel Forward, caaataggcctcttctctttggaacctcaggaacattc,
- HAdel Reverse, gaatgttcctgaggttccaaagagaagaggcctatttg.

The recombinant virus was generated by reverse genetics. In general, the eight plasmids were co-transfected into 293T/MDCK co-cultured monolayer for 72 h^45,46^. Supernatants in culture flask were inoculated in embryonated eggs at 35 °C. Hemagglutination test was used for detection of positive viruses. Virus stocks were sequenced for verification, and virus titrations were determined with MDCK cells. The log_10_ TCID_50_/ml was calculated by Reed and Muench method.

The recombinant H7N9/PR8 virus was evaluated as follows:

1. Virus sequencing: confirm whether each gene is correct and knock out of polybasic amino acids on HA.
2. Lethal dose of H7N9/PR8 recombinant virus on chicken embryo.
3. Trypsin dependent test: confirmed that the replication of recombinant H7N9/PR8 virus depends on TPCK trypsin.

### Cells and compounds

The grown and maintainance of MDCK cells and 293 T cells were described before^24^. In short, cells were grown and maintained in Dulbecco’s modified Eagle’s medium (DMEM; Invitrogen) supplemented with 10% fetal bovine serum (FBS, Invitrogen), HEPES (10 mM, Invitrogen), penicillin (100 units/ml), and streptomycin (100 μg/ml, Invitrogen). The NA inhibitors used in vitro experiment were oseltamivir carboxylate (Hoffman-La Roche), zanamivir (GlaxoSmithKline), Peramivir (MCE company) and Laninamivir (MCE company). The pro-drug oseltamivir phosphate (oseltamivir) used in mice was purchased from MCE company. The NA inhibitors were prepared in sterile distilled water and stored in aliquots at − 20 °C.

### NA inhibition test

Inhibition of NA enzyme activity of the 4 NA inhibitors was assessed in the fluorescence-based NA inhibition assay, using the NA-Fluor Influenza Neuraminidase Assay kit (Applied Biosystems, ThermoFisher) as previously described^47^. IC_50_ values, defined as the concentration of drug required to reduce enzyme activity by 50%, were calculated using GraphPad prism5 software. Interpretation of IC_50_ was performed using the WHO Antiviral Working Group (WHO-AVWG) criteria^47–49^: the testing virus was compared with the drug-sensitive reference virus, for influenza A viruses, a < tenfold increase in IC_50_ represents normal inhibition, and a ten to 100-fold increase represents reduced inhibition, while a > 100-fold increase is highly reduced inhibition.

### Growth kinetics in MDCK cells

The method of growth kinetics of HPAI H7N9 viruses in MDCK cells was described before^24^. Briefly speaking, MDCK cells were infected with the recombinant virus at an MOI of 0.001 at 37 °C 5% CO_2_. Cells were washed twice with PBS after 1 h of incubation, and infection medium containing TPCK-trypsin was added. At time points 0, 18, 24, 48, 72 and 96 h post-infection (hpi), supernatants were collected, and 3 biological repeats were set up for each sample. Viral titers were determined on MDCK cells.

### Pathogenicity in mice

All mouse experiments were conducted after the approval of the Ethics Committee of the National Institute for Viral Disease Control and Prevention, China CDC (20191106039). For pathogenic study, specific-pathogen-free (SPF), female 8-week-old C57BL/6 mice were intranasal inoculated with 50 μl of 10^1^–10^6^ TCID_50_ recombinant H7N9/PR8 virus under isoflurane anaesthesia^50^. The weight change from 1 to 14 days post-infection (dpi) was calculated as a percentage of weight on 0 dpi (n = 5/group). Mice that showed severe disease and lost ≥ 20% of initial weight were euthanized. On 1, 3, 5, 7 and 14 dpi after inoculation, the lungs and trachea were removed from 3 mice of each group infected with 10^4^–10^5^ TCID_50_ virus, rinsed with sterile phosphate-buffered saline (PBS), homogenized, and resuspended in 1 ml of PBS. The suspensions were cleared by centrifugation at 3000×*g* for 20 min, and virus yield was determined by TCID_50_ in MDCK cells. On 3dpi, Lungs of mice infected with 10^4^–10^5^ TCID_50_ virus (n = 3/group), were fixed in 10% paraformaldehyde and then for pathological study.

### Oseltamivir efficacy in mice

SPF, female 8-week-old C57BL/6 mice were intranasal inoculated with 50 μl of two 50% mouse lethal doses (MLD_50_) of recombinant H7N9/PR8 viruses under isoflurane anaesthesia. Starting 0 dpi, mice in the drug treatment group were orally administered 100 μl oseltamivir at the dosage of 20 mg/kg/day BID (at 12 h intervals) for 5 days^16^. Untreated mice orally received 100 μl PBS twice daily for 5 days. Body weights and survival of infected mice were monitored for 14 days daily (n = 5/group). Mice that lost more than 20% of their initial weight were euthanized. The body weight change of mice was calculated and shown as a percentage of its initial weight. On 2, 4 and 6 dpi, the lungs were removed from 3 mice per group for viral load titration and pathological study.

### Lung histopathology and immunohistochemistry

Pathological research methods were described in previous studies^49^. In short, the lung tissue of mice fixed with formalin was cut into sections and then stained with hematoxylin and eosin (H&E). The distribution of antigens (influenza A nucleoprotein) was studied by immunohistochemistry (IHC).

### Serologic tests

Hemagglutination inhibition (HI) assay test was done to calculate the mouse infective dose of 50% animals (MID_50_)^49^. In short, at 14 dpi, serum samples were obtained from mice that survived from the pathogenic study. Sera were collected by retro-orbital bleed, treated with receptor-destroying enzyme for 18 h, and then heat-inactivated at 56 °C for 1 h, and tested by HI assay with 1% turkey red blood cells. HA titer ≥ 40 is positive; HA titer < 40 is negative.

### Crystal structure display of NA substitutions

The existing NA protein crystal structure of A/Anhui/1/2013 H7N9 virus (PDB ID: 4MWJ)^37^ in Protein Data Bank (PDB) was used, based on which a crystal structure model of the NA monomer was obtained. DeepViewv4.1.0 software was used to display and label drug resistance mutations.

### Statistical analysis

NAIs susceptibility and viral replication were analysis by the GraphPad Prism 5.0 software. Student’s t test was used to evaluate differences between two groups. ANOVA test and Kruskal–Wallis H test were used to evaluate differences among four groups. Cox regression was used to analyze hazard ratio by SPSS software. A *P* value of < 0.05 was considered significant.

### Biosafety

All experiments involving HPAI H7N9 viruses were operated by qualified person in BSL-3 laboratory of National Institute for Viral Disease Control and Prevention, Chinese center for disease control and prevention.

### Statement of the ARRIVE guidelines

We confirmed the study was carried out in compliance with the ARRIVE guidelines.

## Supplementary Information

Supplementary Figure S1.

Supplementary Table S1.

Supplementary Table S2.
