# Supplementary material for: Substitution of I222L-E119V in neuraminidase from highly pathogenic avian influenza H7N9 virus exhibited synergistic resistance effect to oseltamivir in mice
Source: Sci Rep. 2021 Aug 11;11:16293. doi: 10.1038/s41598-021-95771-4 (PMC8358046; doi:10.1038/s41598-021-95771-4)
Supplement: Supplementary file 1 — Supplementary Figure S1. [file 41598_2021_95771_MOESM1_ESM.pdf]

Substitution of I222L-E119V in neuraminidase from highly pathogenic avian influenza H7N9 virus exhibited synergistic resistance effect to oseltamivir in mice

Jing Tang, Rongbao Gao, Liqi Liu, Qiongqiong Fang, Zhaomin Feng, Jia Liu, Xiyan li, Dayan Wang

National Institute for Viral Disease Control and Prevention, Chinese Center for Disease Control and Prevention; WHO Collaborating Center for Reference and Research on Influenza, Beijing, China

*Corresponding author: Dayan Wang, National Institute for Viral Disease Control and Prevention, Chinese Centers for Disease Control and Prevention; WHO Collaborating Center for Reference and Research on Influenza, No.155 Changbai road, Changping district, Beijing, China. Phone: 010-58900850; dayanwang@cnic.org.cn

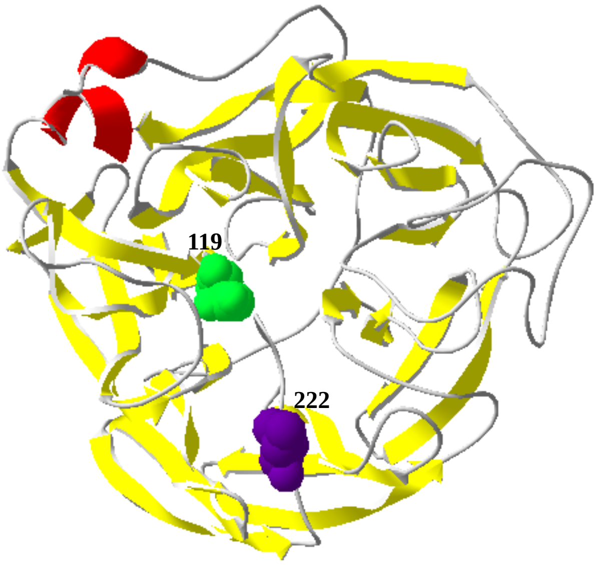

Supplementary figure1. Crystal structure display of E119V-I222L in NA of H7N9 virus. Crystal structure model of NA monomer of A/ Anhui1/2013/H7N9 virus (PDB ID: 4MWJ) was used to display and label the two mutations (E119V and I222L) by DeepView v4.1.0 software. E119Vin NA was showed in green ball; I222L in NA was showed in purple ball.
