# Supplementary material for: Substitution of I222L-E119V in neuraminidase from highly pathogenic avian influenza H7N9 virus exhibited synergistic resistance effect to oseltamivir in mice
Source: Sci Rep. 2021 Aug 11;11:16293. doi: 10.1038/s41598-021-95771-4 (PMC8358046; doi:10.1038/s41598-021-95771-4)
Supplement: Supplementary file 2 — Supplementary Table S1. [file 41598_2021_95771_MOESM2_ESM.docx]

| **Supplementary table1 Generation of recombinant viruses** | | | | |
| --- | --- | --- | --- | --- |
| NO. | HA gene | NA gene | Internal genes | RG virus'（E1）name |
|  |  |  |  |  |
| 1 | 006 HA | 006 NA 222I-119E | 006 6 internal genes | rg006NA |
| 2 | 006 HA | 006 NA 222L | 006 6 internal genes | rg006NA222L |
| 3 | 006 HA | 006 NA 119V | 006 6 internal genes | rg006NA119V |
| 4 | 006 HA | 006 NA 222L-119V | 006 6 internal genes | rg006NA222L-119V |
| 5 | 006HAdel | 006 NA 222I-119E | PR8 6 internal genes | rg006NA/PR8 |
| 6 | 006HAdel | 006 NA 222L | PR8 6 internal genes | rg006NA222L/PR8 |
| 7 | 006HAdel | 006 NA 119V | PR8 6 internal genes | rg006NA119V/PR8 |
| 8 | 006HAdel | 006 NA 222L-119V | PR8 6 internal genes | rg006NA222L-119V/PR8 |
