# Supplementary material for: Substitution of I222L-E119V in neuraminidase from highly pathogenic avian influenza H7N9 virus exhibited synergistic resistance effect to oseltamivir in mice
Source: Sci Rep. 2021 Aug 11;11:16293. doi: 10.1038/s41598-021-95771-4 (PMC8358046; doi:10.1038/s41598-021-95771-4)
Supplement: Supplementary file 3 — Supplementary Table S2. [file 41598_2021_95771_MOESM3_ESM.docx]

| **Supplementary table2 Serum antibody titer of C57 mice infected with H7N9/PR8 recombinant viruses** | | | | | | |
| --- | --- | --- | --- | --- | --- | --- |
| Virus and dose | HI titer(rg006NA/PR8 antigen) | | | | | MID50, |
| log10TCID50/50μl | Mouse1 | Mouse2 | Mouse3 | Mouse4 | Mouse5 | log10TCID50/50μl |
| rg006NA/PR8 |  |  |  |  |  | 0.83 |
| 6 | 160 | ND | ND | ND | ND |  |
| 5 | 160 | 160 | ND | ND | ND |  |
| 4 | 160 | 160 | 80 | 80 | 80 |  |
| 3 | 160 | 80 | 80 | 80 | 80 |  |
| 2 | 160 | 80 | 80 | 80 | 80 |  |
| 1 | 40 | 40 | 40 | 20 | 20 |  |
| rg006NA119V/PR8 |  |  |  |  |  | 1 |
| 6 | 160 | ND | ND | ND | ND |  |
| 5 | 320 | ND | ND | ND | ND |  |
| 4 | 320 | 160 | 160 | 80 | 80 |  |
| 3 | 80 | 80 | 80 | 80 | 80 |  |
| 2 | 160 | 160 | 80 | 80 | 10 |  |
| 1 | 160 | 80 | 80 | 20 | 20 |  |
| rg006NA222L/PR8 |  |  |  |  |  | 0.83 |
| 6 | ND | ND | ND | ND | ND |  |
| 5 | ND | ND | ND | ND | ND |  |
| 4 | 160 | 320 | 320 | 160 | ND |  |
| 3 | 160 | 160 | 160 | 160 | 160 |  |
| 2 | 160 | 160 | 160 | 80 | 160 |  |
| 1 | ＜10 | ＜10 | 40 | 160 | 160 |  |
| rg006NA222L-119V/PR8 |  |  |  |  |  | 1.38 |
| 6 | ND | ND | ND | ND | ND |  |
| 5 | ND | ND | ND | ND | ND |  |
| 4 | 160 | 80 | 80 | 80 | 80 |  |
| 3 | 160 | 160 | 80 | 80 | 80 |  |
| 2 | 160 | 80 | 80 | 80 | 80 |  |
| 1 | 80 | 20 | 20 | 20 | 10 |  |
| PBS | ＜10 | ＜10 | ＜10 | ＜10 | ＜10 |  |

ND: not detected.

HI ≥ 40 is positive, HI < 40 is negative.
